# Novel Genetically Modified Mouse Model to Assess Soman-Induced Toxicity and Medical Countermeasure Efficacy: Human Acetylcholinesterase Knock-in Serum Carboxylesterase Knockout Mice

**DOI:** 10.3390/ijms22041893

**Published:** 2021-02-14

**Authors:** Brenda M. Marrero-Rosado, Michael F. Stone, Marcio de Araujo Furtado, Caroline R. Schultz, C. Linn Cadieux, Lucille A. Lumley

**Affiliations:** 1 Medical Toxicology Research Division, US Army Medical Research Institute of Chemical Defense, Aberdeen Proving Ground, MD 21010, USA; brenda.m.marrero-rosado.ctr@mail.mil (B.M.M.-R.); michael.f.stone24.civ@mail.mil (M.F.S.); caroline.r.schultz.civ@mail.mil (C.R.S.); christena.l.cadieux.civ@mail.mil (C.L.C.); 2 Anatomy, Physiology and Genetics Department, Uniformed Services University of the Health Sciences, Bethesda, MD 20814, USA; marcio.furtado@usuhs.edu; 3 BioSEaD, LLC, Rockville, MD 20850, USA

**Keywords:** chemical warfare nerve agent, C57BL/6-Ces1c*^tm1.1Loc^*AChE*^tm1.1Loc^*/J, midazolam, ketamine, soman, GD, mice

## Abstract

The identification of improved medical countermeasures against exposure to chemical warfare nerve agents (CWNAs), a class of organophosphorus compounds, is dependent on the choice of animal model used in preclinical studies. CWNAs bind to acetylcholinesterase and prevent the catalysis of acetylcholine, causing a plethora of peripheral and central physiologic manifestations, including seizure. Rodents are widely used to elucidate the effects of CWNA-induced seizure, albeit with a caveat: they express carboxylesterase activity in plasma. Carboxylesterase, an enzyme involved in the detoxification of some organophosphorus compounds, plays a scavenging role and decreases CWNA availability, thus exerting a protective effect. Furthermore, species-specific amino acid differences in acetylcholinesterase confound studies that use oximes or other compounds to restore its function after inhibition by CWNA. The creation of a human acetylcholinesterase knock-in/serum carboxylesterase knockout (C57BL/6-Ces1c*^tm1.1Loc^*AChE*^tm1.1Loc^*/J; a.k.a KIKO) mouse may facilitate better modeling of CWNA toxicity in a small rodent species. The current studies characterize the effects of exposure to soman, a highly toxic CWNA, and evaluate the efficacy of anti-seizure drugs in this newly developed KIKO mouse model. Data demonstrate that a combination of midazolam and ketamine reduces seizure duration and severity, eliminates the development of spontaneous recurrent seizures, and protects certain brain regions from neuronal damage in a genetically modified model with human relevance to organophosphorus compound toxicity. This new animal model and the results of this study and future studies using it will enhance medical countermeasures development for both defense and homeland security purposes.

## 1. Introduction

The use of rodents as small animal models for toxicological studies is common for reasons that include the high throughput collection of data, relatively lower costs, and most importantly the availability of a wide selection of genetically modified rodents that allow for, among other purposes, a more accurate prediction of response in humans. In the field of toxicology, it is critical to identify more effective treatments against the toxic effects of acute poisoning to organophosphorus (OP) compounds. Chemical warfare nerve agents (CWNAs), a class of OP compounds that are chemically related to pesticides used worldwide, are of particular interest due to their extremely high toxicity, ease of access by nefarious groups and potential for mass casualties. Recent uses of the inhalation threat agent sarin in Syria and the contact hazards VX in Malaysia and fourth-generation agents in the United Kingdom and Russia demonstrate the lethal and incapacitating effects of these agents. Acute poisoning with CWNAs results in the accumulation of the neurotransmitter acetylcholine due to the inhibition of acetylcholinesterase (AChE), an enzyme responsible for its catalysis in neuronal synapses during the process of neurotransmission. The acute physiological consequences of exposure to CWNAs may include severe seizures progressing to status epilepticus (SE), generalized muscular tonus, cardiovascular and respiratory complications, and death, depending on the dose and expediency of medical countermeasures. Surviving victims of CWNA attacks suffer long-lasting repercussions of the acute toxic insult that includes cognitive and psychiatric impairments [1,2]. Survivors of the Tokyo subway sarin attack have persistent brain morphological changes, the severity of which is correlated to the levels of AChE activity present in serum on the day of exposure [3]. Additional neurological signs, as well as post-traumatic stress disorder and anxiety, have also been reported in longitudinal studies of victims of acute OP poisoning (reviewed in [4,5]). The identification of improved medical countermeasures that can aid in the prompt recovery of CWNA exposure victims, whether warfighters or civilians, relies heavily on information acquired from pre-clinical studies. Thus, it is imperative to elucidate the neuropathological and behavioral changes over time following acute exposure to CWNA in preclinical models that mimic human response.

Rodents are widely used in preclinical research to study the acute, sub-chronic, and long-term effects of exposure to CWNA doses that lead to prolonged SE. Studies in our laboratory and those of others have shown that rats exposed to soman (GD), an extremely toxic CWNA, show cognitive deficits and neuropathological consequences even when medical countermeasures are administered [6,7,8,9,10]. Similar to the effects observed in rats, neuropathology and behavioral deficits also occur in guinea pigs in the days and weeks after acute GD exposure [11]. In addition to performance deficits and neuropathological effects of GD exposure, our laboratory was the first to quantify the development of epileptogenesis in rats and in carboxylesterase (CaE) knockout (Es1-/-) mice exposed to GD [12,13]. Although rats and mice are traditionally used to evaluate medical countermeasures against OP chemical-induced toxicity, a crucial confounder has been the presence of plasma CaE activity, which is not present in humans. Carboxylesterase, an enzyme involved in the detoxification of certain OP compounds, including GD, serves as an endogenous scavenger of the compound and reduces the concentration of the OP that is freely available to inhibit AChE [14], thereby providing some protection [15]. This species difference is eliminated in the Es1-/- mouse that specifically lacks CaE activity in plasma, while maintaining its activity in tissues [16]. As expected, the median lethal dose (LD50) of GD and other OP chemicals is lower in Es1-/- mice compared to wild-type (C57BL/6) mice [13,17,18]. Our laboratory established a GD exposure model in Es1-/- mice in which high doses were required to reliably induce electroencephalographic (EEG) seizure activity and associated neuronal loss and neuroinflammatory response [13]. More recently, researchers at the U.S. Army Medical Research Institute of Chemical Defense (USAMRICD) cross-bred the Es1-/- mouse strain with another mouse strain in which the mouse gene that expresses AChE was replaced with the human AChE gene, resulting in the human AChE knock-in/CaE knockout (C57BL/6-Ces1c^*tm1.1Loc*^AChE^*tm1.1Loc*^/J; a.k.a KIKO) mouse strain [19,20]. The creation of the KIKO mouse strain makes it possible to more accurately predict the human response to the CWNA and other OP compounds in a small animal model and to better evaluate medical countermeasures against their effects for the protection or treatment of warfighters and civilians.

The majority of research on medical chemical defense has focused on characterizing the acute lethal effects of CWNA exposure and the discovery of medical countermeasures to promote survival against lethal doses in animal models when treatment is administered prior to exposure or shortly thereafter. However, delayed treatment with medical countermeasures is likely in various CWNA attack scenarios on unprepared populations and/or mass casualties (defense or civilian), as well as in cases when the toxic agent is unknown. Current treatments against CWNA exposure include atropine sulfate as a muscarinic acetylcholine receptor antagonist, pralidoxime (a.k.a. 2-PAM) as an oxime to reactivate CWNA-bound AChE, and a benzodiazepine (diazepam or midazolam) as an anti-seizure drug. Although these promote survival, they are not fully protective of function in rodent models when their administration is delayed or in response to extremely high agent doses. In particular, the successful treatment of cholinergic-induced seizure with benzodiazepines is inversely correlated with seizure duration and brain pathology [9,21,22], further supporting the need for treatments that work either as an adjunct to or downstream of first-line anti-seizure treatment after an acute CWNA exposure. Even when seizures seem to stop by the administration of diazepam at 1 or 2 h after GD exposure, the intermittent return of seizures is observed in rats so that the overall duration of seizures in the first 24 h after the toxic insult is similar to that of animals that did not receive any anti-seizure treatment [23]. Midazolam, recently approved for the treatment of acute repetitive seizures, increases survival in GD-exposed rats and Es1-/- mice but does not prevent the development of spontaneous recurrent seizures (SRS) or brain pathology when treatment is delayed [13,18,24,25,26].

The circumstances that give rise to the refractoriness of prolonged seizures to benzodiazepines include the internalization of γ-aminobutyric acid type A (GABAA) receptors and trafficking of N-methyl-D-aspartate (NMDA) receptors to the surface of neurons, which promote reduced neuronal inhibition and increased excitation [27,28]. It is, therefore, of great benefit for the treatment of prolonged status epilepticus to include a combination of therapies that target the maladaptive changes in receptor composition that occur in neurons during a cholinergic-induced seizure. Our laboratory and those of others have demonstrated the improved anti-seizure effects of adding ketamine, an NMDA receptor antagonist, to a benzodiazepine treatment in animal models of pilocarpine- and CWNA-induced status epilepticus [29,30,31,32]. Others have also shown the effectiveness of ketamine in combination with atropine sulfate in halting GD-induced seizure, preventing lethality, and providing neuroprotection as a delayed treatment in guinea pigs [33,34]. Similar anti-seizure and neuroprotective effects have been observed against lithium-pilocarpine-induced seizures in rats that were administered ketamine at 15 min (min) after onset [35]. In the Es1-/- mouse, our laboratory has shown that in addition to increasing survival and reducing seizure severity and incidence of epileptogenesis, the combination of midazolam with ketamine administered at 40 min after seizure onset was able to reduce neurodegeneration and neuroinflammation in brain regions associated with seizure neuropathology [31]. Therefore, we hypothesized that the addition of ketamine to a delayed midazolam treatment would further validate the benefits of the dual-therapy in reducing epileptogenesis and brain pathology following GD-induced seizure in the KIKO mouse model. We suggest the KIKO model to be a useful preclinical animal model to predict effects in humans and that this model will prove useful in the advancement of improved medical countermeasures against CWNA exposure.

## 2. Results

The present study included two main objectives: (1) the development of a GD exposure paradigm that would elicit in KIKO mice seizures that do not respond to a midazolam treatment while maintaining relatively high survival, and (2) the evaluation of the efficacy of ketamine as an adjunct to delayed midazolam treatment on seizure severity, survival, and neuropathological effects of GD exposure.

### 2.1. Development of GD Exposure Paradigm in KIKO Mice

#### 2.1.1. High-Dose GD Exposure Rapidly Induced Seizure in KIKO Mice; Delayed Midazolam Treatment Did Not Halt Seizure Activity

**Figure 1:**
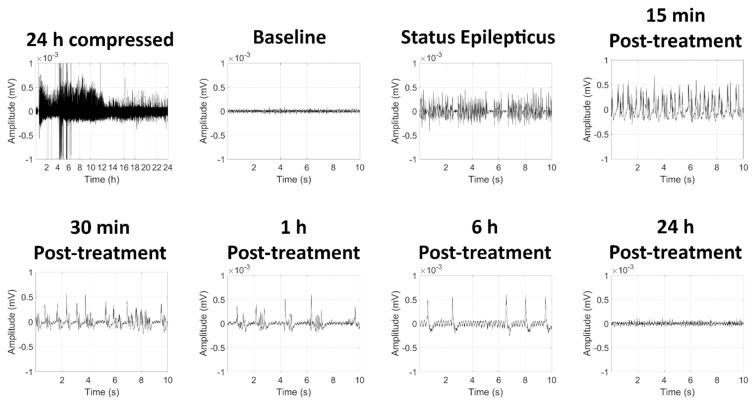
Effects of soman-induced status epilepticus and delayed midazolam treatment on electroencephalographic (EEG) activity in KIKO mice. Representative images are shown of 24 h compressed signal and 10-sec recordings at baseline (24 h prior to soman exposure), status epilepticus, as well as 15 min, 30 min, 1 h, 6 h, and 24 h after midazolam (3 mg/kg; SC) treatment.

Following SC exposure to GD, KIKO mice exhibited toxic signs that included whole-body tremors and convulsions. Running seizures (tremors while walking rapidly) developed before the appearance of high-amplitude spiking in EEG recording that is indicative of seizure activity. Representative images of the EEG signal from a GD-exposed mouse are shown in Figure 1. The electroencephalographic activity was monitored in real-time, and midazolam was administered 15 min after the onset of SE. Synchronous high-amplitude peaks in EEG signal could still be observed at 15 min, 30 min, 1 h, and 6 h following midazolam treatment, suggesting that seizure did not respond to the human equivalent dose of midazolam.

#### 2.1.2. GD Exposure Resulted in Robust Neurodegeneration and Neuroinflammation in Brains of KIKO Mice at 24 h Following GD Exposure and Delayed Midazolam Treatment

**Figure 2:**
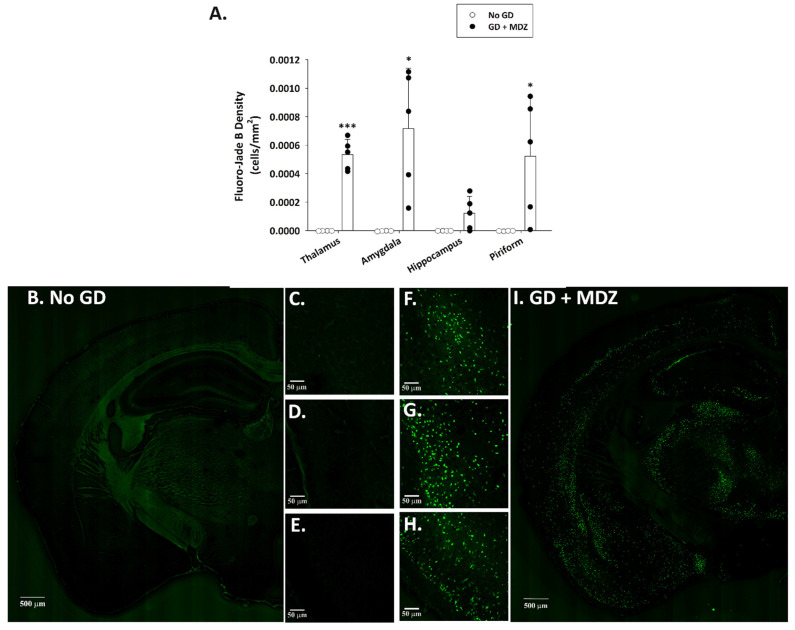
Effect of soman-induced status epilepticus and delayed midazolam (MDZ; 3 mg/kg; SC) treatment on neuronal cell degeneration in KIKO mice at 24 h after exposure. Soman-exposed (SC) KIKO mice were treated with midazolam at 15 min after seizure onset. At 24 h after saline or soman (GD) exposure, mice were perfused and brains were collected for staining with Fluoro-Jade B to visualize dying neurons. (**A**) Fluoro-Jade B-positive cell density was estimated in the thalamus, amygdala, hippocampus, and piriform cortex of GD-exposed mice given midazolam (GD + MDZ; *n* = 5), and no agent controls (No GD; *n* = 4). Representative images taken with a 0.40 NZ 10X objective are shown from (**B**–**E**) control mice and (**F**–**I**) soman-exposed mice. Close-ups of the (**C**,**F**) thalamus, (**D**,**G**) amygdala, and (**E**,**H**) piriform cortex are also shown. * *p* < 0.05, *** *p* < 0.001, compared to No GD control group.

**Figure 3:**
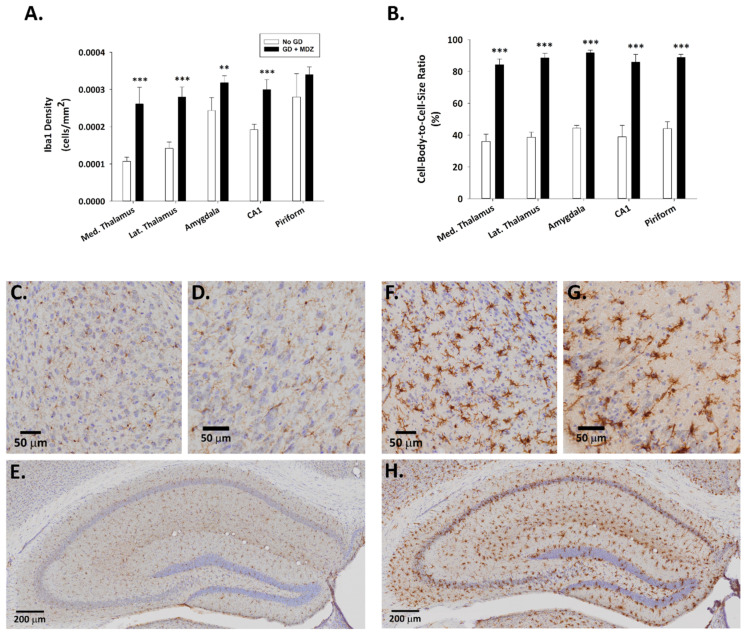
Effect of soman-induced status epilepticus and delayed midazolam (3 mg/kg; SC) treatment on microgliosis and microglial activation in KIKO mice. Soman-exposed (SC) KIKO mice were treated with midazolam 15 min after seizure onset. At 24 h after saline or GD exposure, mice were perfused and brains were collected for immunohistochemical processing with and antibody against Iba1 to visualize microglia. Cresyl violet was used for the localization of anatomical landmarks. Measures of (**A**) cell density and (**B**) cell-body-to-cell-size ratio were estimated in the medial (Med.) thalamus, lateral (Lat.) thalamus, amygdala, amygdala, CA1 region of the hippocampus, and layer 3 of the piriform cortex of GD-exposed mice given midazolam (GD + MDZ; *n* = 5) and no agent controls (No GD; *n* = 4). Representative images taken with a 0.75 NA 20X objective are shown from (**C**–**E**) control mice and (**F**–**H**) soman-exposed mice. Close-ups of the (**C**,**F**) amygdala, (**D**,**G**) piriform cortex, and (**E**,**H**) hippocampus are shown. ** *p* < 0.01, *** *p* < 0.001, compared to No GD control group.

Fluoro-Jade B staining, extensively used as a reliable method for detecting degenerating neurons in the brain, was not observed in control animals (Figure 2). Conversely, a significantly higher density of Fluoro-Jade B-positive neurons was present in the thalamus, amygdala, and piriform cortex of GD-exposed mice. Brain tissue from GD-exposed animals that was immunoprocessed for Iba1 showed a significantly higher density of microglia in the medial thalamus, lateral thalamus, amygdala, CA1 region of the hippocampus, and the piriform cortex (Figure 3). In the same regions where microglia cell density was elevated, changes in the morphology of cells, indicative of a reactive state, were observed in the GD-exposed mice (Figure 3).

### 2.2. Evaluation of Ketamine as an Adjunct to Delayed Midazolam Treatment in GD-Exposed KIKO and Es1-/- Mice

#### 2.2.1. Ketamine Increased Survival in GD-Exposed KIKO and Es1-/- Mice Treated with Midazolam

**Figure 4:**
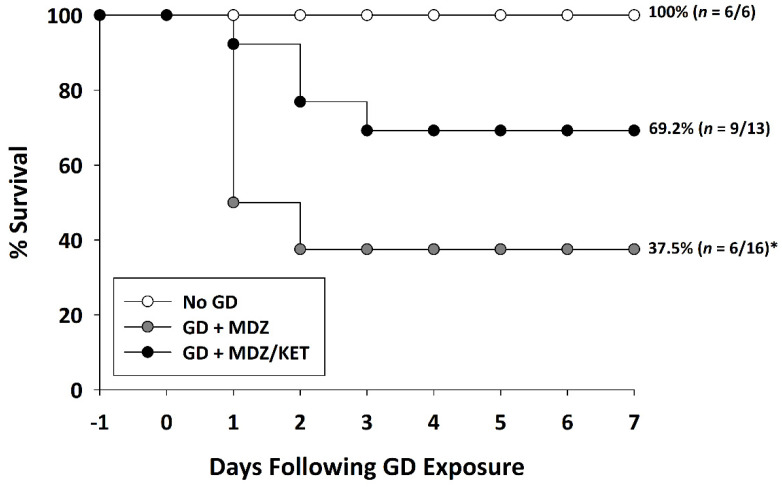
Effect of delayed midazolam (MDZ) treatment combined with ketamine (KET) on survival following GD-induced status epilepticus in KIKO mice. Mice exposed to GD and treated with midazolam monotherapy (GD + MDZ) at 15 min after seizure onset had a significantly lower percentage of survival compared to that of the saline control (No GD) group. In contrast, soman-exposed mice that were treated with a combination of midazolam and ketamine (GD + MDZ/KET) had a higher percentage of survival that was not significantly different from the percentage of survival of the No GD group. * *p* < 0.05, compared to No GD group.

KIKO and Es1-/- mice were exposed (SC) to saline or 80 µg/kg of GD and administered delayed anti-seizure treatment that consisted of either midazolam mono-therapy or a midazolam/ketamine combination therapy. Following exposure, the survival of animals was monitored over the course of 7 days (Figure 4). A Chi-squared analysis followed by a Fisher’s exact test revealed that the percentage of KIKO mice surviving at the endpoint in the midazolam mono-therapy group (37.5%) was significantly reduced compared with that in the No GD group (100%). Percent survival in the KIKO midazolam/ketamine combination therapy group (69.2%) was not significantly different from that in the No GD group survival. Similarly, in the Es1-/- mice the percentage of mice surviving at the endpoint (14.3%) was significantly reduced compared to that in the No GD group (100%), while the percent of survival in the midazolam/ketamine group (55.6%) was not significantly different from No GD control (Figure S1).

#### 2.2.2. Ketamine in Combination with Midazolam at 15 Min after Seizure Onset Significantly Reduces Behavioral Seizure Severity, Acute Seizure Duration, and Changes in EEG Power in GD-Exposed KIKO Mice, and Prevents the Development of Spontaneous Recurrent Seizures

**Figure 5:**
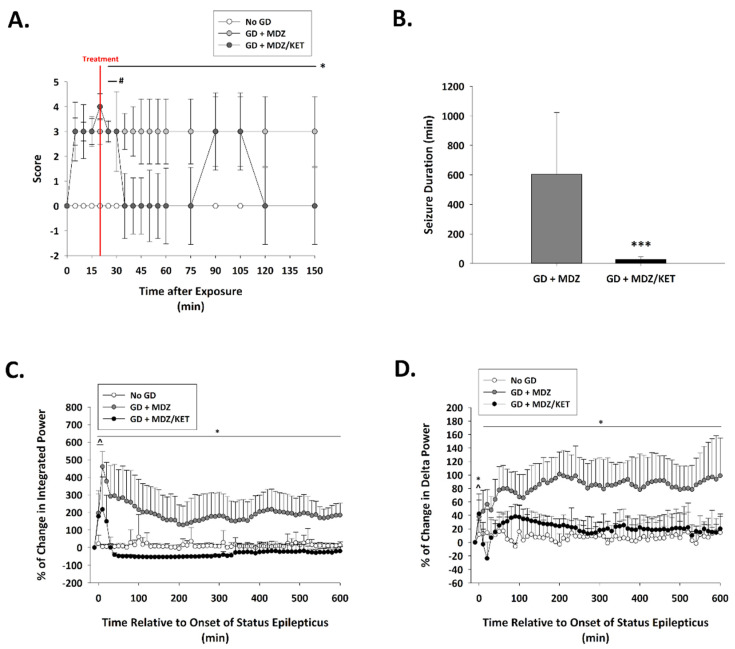
Effect of ketamine as an adjunct to delayed midazolam treatment on seizure activity and severity in soman-exposed KIKO mice. (**A**) SC exposure to 80 μg/kg of GD resulted in the appearance of behavioral seizure signs within 5 min of exposure. The severity of toxic signs was scored following a modified Racine scale: 0, no abnormality; 1, mastication, tongue fasciculations, oral tonus; 2, head nodding and/or tremors; 3, forelimb clonus or tonus, body tremors; 4, rearing with convulsions; and 5, rearing and falling with convulsions. Following treatment administration (indicated by a red line; 15 min after EEG seizure onset), toxic signs for the midazolam/ketamine group (GD + MDZ/KET; *n* = 13) were transiently reduced in severity within 15 min of treatment for up to 40 min after treatment. In contrast, toxic signs in the midazolam monotherapy group (GD + MDZ; *n* = 15) did not subside. * *p* < 0.05, GD + MDZ compared to no agent control (No GD; *n* = 6); # *p* < 0.05, GD + MDZ/KET compared to No GD. (**B**) Soman exposure elicited seizure that had an average duration of (±SD) 705.3 ± 332.4 min in the GD + MDZ (*n* = 13) group, while an average of 29.4 ± 23.4 min was observed in the GD + MDZ/KET (*n* = 13) group. *** *p* < 0.001, GD + MDZ compared to no agent control (No GD); Tracings of average percentages of relative change in (**C**) integrated power (freq. range) and (**D**) delta (0.1–4 Hz) EEG frequency are shown over a period of 600 min (10 h). In KIKO mice, the midazolam/ketamine combination therapy was able to reduce over time the increase in integral power and delta power immediately following administration of treatment. * *p* < 0.05, GD + MDZ (*n* = 7) compared to No GD (*n* = 5) group; ^ *p* < 0.05, GD + MDZ/KET (*n* = 5) compared to No GD group.

**Figure 6:**
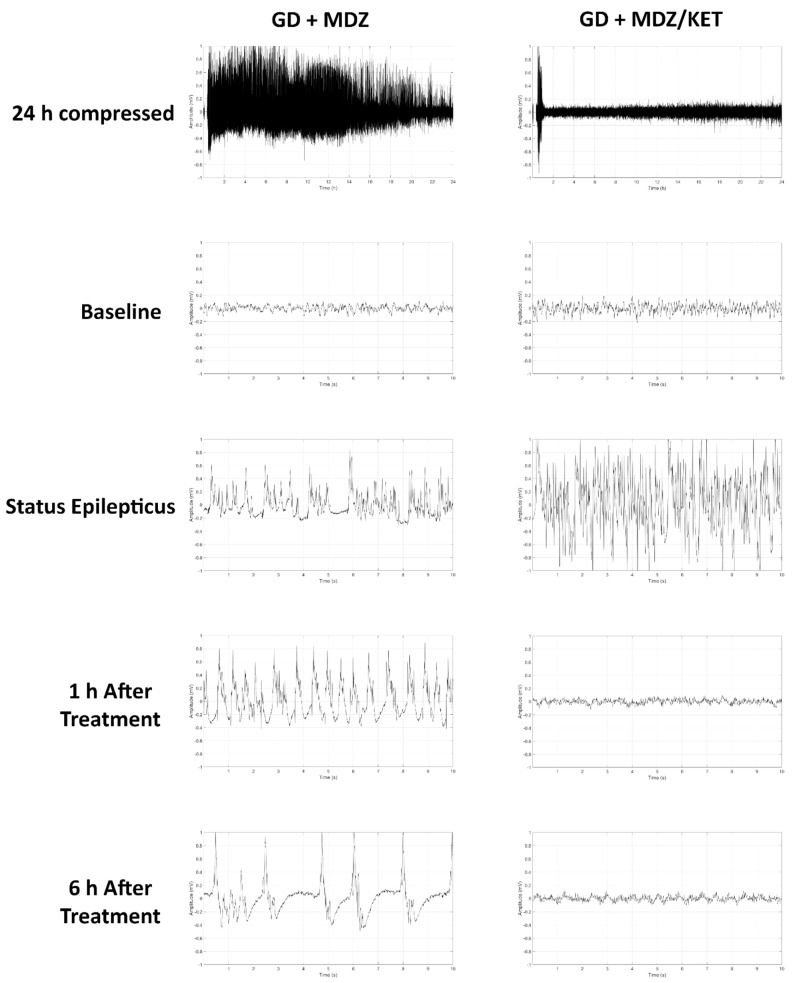
Effects of ketamine as an adjunct to delayed midazolam treatment on soman-induced EEG activity in KIKO mice. Representative images are shown of 24 h compressed signal and 10-sec recordings at baseline (24 h prior to soman exposure), status epilepticus, 1 h after midazolam treatment, and 6 h after midazolam monotherapy (GD+MDZ) or midazolam/ketamine (GD + MDZ/KET) combination therapy.

Exposure to GD resulted in the appearance of severe behavioral seizure signs that included whole-body tremors, forelimb clonus or tonus, and convulsions within 5 min of exposure. In KIKO mice receiving the midazolam ketamine combination, behavioral signs subsided within 15 min of treatment administration; decreased severity of signs lasted for up to 130 min after treatment administration (Figure 5A). In contrast, the severity of behavioral seizures in the midazolam mono-therapy group did not subside in the hours monitored following treatment. Prolonged seizures were elicited in GD-exposed KIKO mice with an average (±SD) latency of 6.6 ± 2.7 min. In mice receiving midazolam mono-therapy seizures lasted an average (±SD) of 603.8 ± 419.5 min in the first 24 h following GD exposure, whereas mice that were treated with a combination of midazolam/ketamine spent an average of 27.5 ± 17.5 min in seizures which was significantly lower compared to the average for midazolam mono-therapy (Figure 5B). GD-induced status epilepticus produced an increase in integrated power immediately after exposure (Figure 5C). Following administration of treatment at 15 min after seizure onset, the integrated power was reduced in mice receiving the midazolam/ketamine combination, whereas those that were administered midazolam mono-therapy continued to have elevated integrated EEG power. Similarly, the power of EEG frequencies in the delta range was reduced following administration of midazolam/ketamine combination therapy, while those mice receiving midazolam mono-therapy continued to show increased delta power throughout the first 10 h after exposure (Figure 5D). The midazolam/ketamine combination treatment was able to promptly halt seizures in KIKO mice, as demonstrated in representative EEG images (Figure 6). Observations in KIKO mice are in agreement with results in male Es1-/- mice that were administered treatment at 15 min after seizure onset. In Es1-/- mice receiving the midazolam ketamine combination behavioral signs subsided within 10 min of treatment administration, while in the midazolam monotherapy group the severity of behavioral seizures did not subside in the hours following treatment (Figure S2A). With an average seizure duration of 70.5 ± 139.2 min, the midazolam/ketamine treatment significantly reduced the duration of the acute GD-induced seizure, whereas in mice receiving midazolam mono-therapy seizures lasted an average (±SD) of 283.2 ± 195.8 min in the first 24 h following GD exposure (Figure S2B), GD exposure resulted in an increase in EEG integral power (Figure S2C) and delta power (Supplemental Figure S2D) that was persistently decreased by midazolam/ketamine treatment.

**Figure 7:**
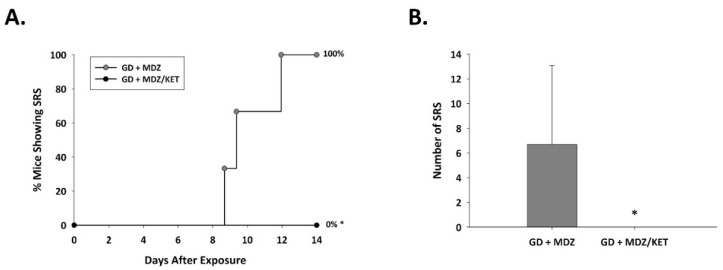
Effect of ketamine as an adjunct to delayed midazolam treatment on the development of spontaneous recurrent seizures in soman-exposed KIKO mice. KIKO mice were SC exposed to soman and treated at 15 min after seizure onset with midazolam monotherapy (GD + MDZ) or midazolam/ketamine combination therapy (GD + MDZ/KET), and EEG activity was monitored for 14 days after exposure. (**A**) The onset of the first detected SRS for each surviving animal is graphed to indicate the percentage of mice in each group that developed SRS. All 3 GD + MDZ mice that survived 14 days after GD-induced seizure developed SRS, whereas 9 survivors of the GD + MDZ/KET group did not develop SRS. (**B**) The average (±SD) number of SRS events is graphed for each group. * *p* < 0.05.2.2.3. Ketamine in Combination with Midazolam at 15 Min after Seizure Onset Offers Neuroprotection and Reduced Neuroinflammatory Response in GD-Exposed KIKO Mice.

In the midazolam mono-therapy group, all three instrumented animals that survived up to 14 days after GD-induced seizure developed SRS following the toxic insult (Figure 7); in this group, the average (±SD) number of SRS events was 6.7 ± 6.4. In contrast, none of the nine surviving mice that were exposed to GD and treated with a midazolam/ketamine combination therapy developed spontaneous recurrent seizures.

**Figure 8:**
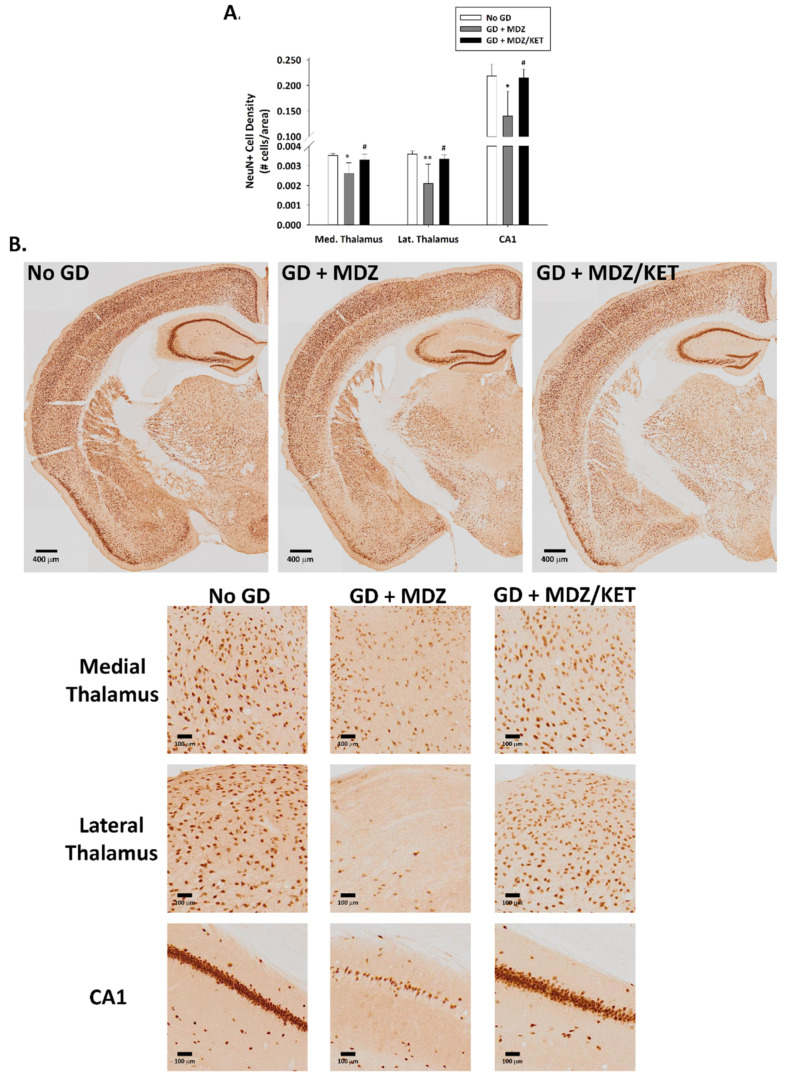
Effect of soman-induced status epilepticus and delayed midazolam monotherapy or midazolam/ketamine combination therapy on neuronal cell population in KIKO mice. At 2 weeks after SC soman-induced status epilepticus and delayed midazolam (GD + MDZ) or midazolam/ketamine (GD + MDZ/KET) therapy, KIKO mice were perfused, and brains collected for immunohistochemical processing with an antibody against NeuN, a neuronal nuclear protein, to visualize mature neurons. (**A**) NeuN-positive cells were counted and cell densities estimated in the medial thalamus, lateral thalamus, amygdala, piriform cortex, and CA1 region of the hippocampus from no agent controls (No GD; *n* = 4), GD + MDZ (*n* = 3), and GD + MDZ/KET (*n* = 5). Using stereology, loss of neurons was also confirmed in the CA1 region of the hippocampus. (**B**) Representative images, taken with a 0.40 NA 10X objective, are shown. * *p* < 0.05, ** *p* < 0.01, compared to No GD group; # *p* < 0.05, compared to GD + MDZ.

**Figure 9:**
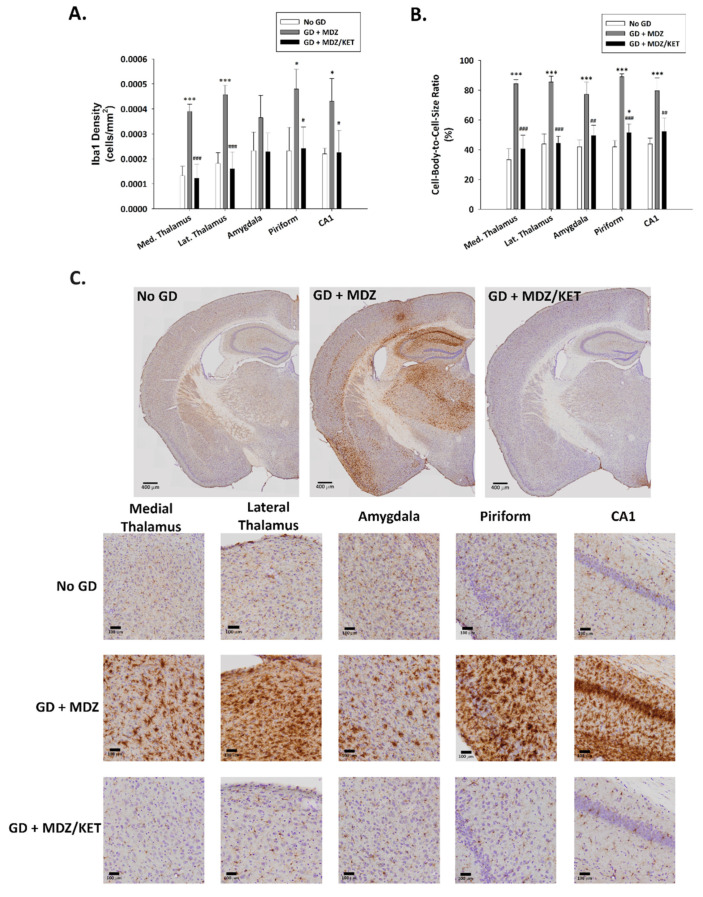
Effect of soman-induced status epilepticus and delayed midazolam monotherapy or midazolam/ketamine combination therapy on microgliosis and microglial cell activation in KIKO mice. At 2 weeks after SC soman-induced status epilepticus and delayed midazolam (GD + MDZ) or midazolam/ketamine (GD + MDZ/KET) therapy, KIKO mice were perfused, and brains collected for immunohistochemical processing with an antibody against Iba1 to visualize microglia. (**A**) Iba1-positive cells were counted and cell densities estimated in the medial thalamus, lateral thalamus, amygdala, piriform cortex, and CA1 region of the hippocampus from no agent controls (No GD; *n* = 4), GD + MDZ (*n* = 3), and GD + MDZ/KET (*n* = 5). (**B**) In the same brain regions, the cell-body-to-cell-size ratio, a measure of the conformational changes that occur following activation of microglia, was estimated. (**C**) Representative images, taken with a 0.75 NA 20X objective, are shown. * *p* < 0.05, *** *p* < 0.001, compared to No GD group; # *p* < 0.05, ## *p* < 0.01, ### *p* < 0.001, compared to GD + MDZ.

A significant effect of treatment on neuronal cell density was observed. Neuronal cell loss, indicated by a reduced density of cells that were immunopositive for the neuronal marker NeuN, was observed at two weeks after GD exposure in the medial thalamus, lateral thalamus, amygdala, piriform cortex, and hippocampal CA1 region of mice that received midazolam mono-therapy (Figure 8). On the other hand, neuronal cell density in mice receiving the midazolam/ketamine combination treatment was not significantly different from that in the No GD control group and significantly higher than that in the midazolam mono-therapy group, indicating that the dual-therapy was able to protect against neuronal cell loss in these regions. Representative images of brain slices immunohistochemically processed for NeuN are shown in Figure 8B. A robust neuroinflammatory response, characterized by the increase in microglial cell density (Figure 9A) and an increase in cell-body-to-cell-size ratio (Figure 9B) was also observed in the medial thalamus, lateral thalamus, piriform cortex, and CA1 region of the hippocampus of GD-exposed animals that received midazolam monotherapy treatment at 15 min after seizure onset. In contrast, KIKO mice that were administered a midazolam/ketamine combination therapy showed microglial densities and cell-body-to-cell-size ratios that were not significantly different from No GD control group. Representative images of brain slices immunohistochemically processed for Iba1 are shown in Figure 9C.

## 3. Discussion

In the current study, we show the acute and sub-chronic effects of GD-induced status epilepticus in the KIKO genetically modified mouse model. Furthermore, using this novel model, we demonstrate the anti-seizure and neuroprotective benefits of adding ketamine as an adjunct to midazolam treatment, in agreement with previous findings in other animal models [29,30,31]. In experiment 1, a GD exposure paradigm resulted in prolonged seizure activity, as demonstrated by the behavioral (e.g., running seizure) and EEG abnormalities, as well as neuronal cell degeneration and neuroinflammation in the first 24 h after exposure. The observations of neuropathology at 24 h after GD-induced seizure in the KIKO mouse are consistent with studies in rats in which neuropathological effects occur within 24 h after GD exposure when the seizure is not quickly terminated by anti-seizure drug administration [9]. The secretion of neurotoxic cytokines, some expressed by microglia, occurs in GD-exposed rats over the acute phase following exposure; peak expression of neuroinflammatory cytokines can be observed at 12–24 h after GD exposure [36]. Thus, the observation that KIKO mice have an increase in microgliosis and microglial activation at 24 h after GD-induced status epilepticus falls in line with the previous observations in other rodent models. Pharmacological intervention with compounds that show anti-inflammatory properties have demonstrated the neurodegenerative role of glial cytokines, including tumor necrosis factor-α (TNF-α), interleukin-1β (IL-1β), and interleukin-6 (IL-6), following OP poisoning. However, it is important to mention that the secretion of other microglia- and astrocyte-derived factors that promote neurogenesis [37] may also contribute as a restorative effect following OP-induced seizure. Microglia have been implicated in the suppression of excess proliferation of neural stem cells and the inhibition of the formation of abnormal neural circuits by phagocytosis of adult-born granule cells in the epileptic dentate gyrus [38]. Future efforts should study the temporal effect of GD-induced gliosis in the KIKO and Es1-/- mouse models and its role in recovery following acute exposure to organophosphorus compounds.

Following the determination that a seizure-inducing dose of GD resulted in neuronal degeneration and an acute neuroinflammatory response, the objective of the second study was to evaluate the efficacy of ketamine as an adjunct to delayed midazolam in terminating status epilepticus and alleviating or preventing epileptogenesis, and neuropathology in the KIKO mouse model. Ketamine is an NMDA receptor antagonist marketed as an anesthetic in human and veterinary uses, but its anti-seizure and neuroprotective properties have been demonstrated in various animal models (reviewed in [39]). A combination of ketamine and atropine sulfate administered to guinea pigs at repeated time points starting at 30 min after GD exposure protects against lethality and drastically reduces seizure activity [33]. The S(+) isomer of ketamine also confers similar protection against lethality and seizure-related brain damage in guinea pigs [34]. Benefits are also observed when combining benzodiazepines with ketamine for the treatment of cholinergic-induced SE. Treatments of cholinergic-induced SE with combinations of diazepam and ketamine [40,41] or midazolam and ketamine [29,30,31] are effective in reducing the severity of seizures and brain pathology.

An increase in survival, consistent with observations in the Es1-/- mouse [31], was observed in KIKO mice that received a treatment of midazolam/ketamine at 15 min after seizure onset compared to those that were administered midazolam only. Interestingly, in the current study, seizure duration was significantly reduced in animals receiving midazolam/ketamine treatment which is in contrast with our previous data showing that in Es1-/- mice this combination therapy did not alter seizure duration but did reduce seizure severity. Since it was likely that the differences in the two animal models were due to the timing at which the therapy was administered, we included in our current studies a group of Es1-/- mice that were administered the midazolam/ketamine treatment at 15 min after seizure onset. The control of seizure at the earlier time point was observed in both KIKO and Es1-/- mice, thus eliminating the possibility of a strain difference. The earlier treatment with anti-seizure drugs following seizure onset results in more effective treatment, as also demonstrated by others [10], suggesting the clinical importance of early intervention to terminate seizure and reduce long-term effects of prolonged seizure activity.

GD-exposed KIKO and Es1-/- mice that developed prolonged status epilepticus showed an increase in delta as well as integrated power that was consistent with previous observations in the Es1-/- mouse model [13,31] and in rats [42]. In GD-exposed Es1-/- mice, an increase in power spectral density and delta power is observed in the minutes following the onset of seizure even when medical countermeasures are administered [13,31]. A sustained reduction in the integrated power and delta power was observed in KIKO mice that were administered a midazolam/ketamine combination treatment. Previous studies have found a correlation of delta power and neuropathological damage in GD-exposed rats [42,43] and other animal models (reviewed in [44]). In the current study, the prolonged increase in delta observed in the midazolam mono-therapy group could relate to the observation of neuronal cell loss. In contrast, the ability of midazolam/ketamine therapy to reduce the changes in the power of delta may underlie the neuroprotective effect that was observed in this group. The prompt control of seizure activity by the midazolam/ketamine combination therapy is highly likely to have played a crucial role in the lethality and neuropathology outcome. Shih et al. [10] have previously demonstrated a strong correlation between the control of initial seizure and protection against the lethal effects of CWNA exposure, as well as a greater severity of neuropathology in animals whose seizure was not controlled by anti-seizure treatment. Additionally, we have previously shown that in the days following GD-induced status epilepticus some rats develop spontaneous recurrent seizures that are directly correlated to severe neuropathology [12]. The Es1-/- mouse model of GD exposure [13,31], as well as the pilocarpine-induced mouse model of status epilepticus [45], also exhibit the development of spontaneous recurrent seizures. A reduced incidence of epileptogenesis may be another factor that played a role in reducing neuropathology in KIKO mice that received the midazolam/ketamine combination therapy. The anti-epileptic effect was also observed in Es1-/- mice that were treated with midazolam/ketamine at 40 min after GD-induced seizure onset, even in the absence of a significant effect on acute seizure activity [31]. In addition to neuroprotective effects, a reduction in neuroinflammation was also observed in KIKO mice that were administered a midazolam/ketamine combination therapy. Dhote at al. [46] have previously shown that in a mouse model of prolonged GD-induced seizure repeated subanesthetic doses of ketamine combined with atropine sulfate were sufficient to suppress glial activation and reduce the expression of pro-inflammatory cytokines following GD exposure. The neuroinflammatory response, including the production of pro-inflammatory cytokines IL-6 and TNF-α, is also curbed by ketamine treatment following an insult in a traumatic brain injury model [47]. Moreover, pre-treatment with ketamine is also able to reduce the increase in microglia and pro-inflammatory cytokines in the frontal cerebral cortex of a sheep model of fetal transient hypoxia [48]. Thus, the literature is in agreement with our results.

## 4. Materials and Methods

### 4.1. Animals

Male (22–32 g) KIKO (C57BL/6-Ces1c*^tm1.1Loc^*AChE*^tm1.1Loc^*/J) and plasma carboxylesterase (Es1-/-) mice and female (16–27 g) KIKO mice obtained from the USAMRICD breeding colony were exposed to GD at an age of 10–11 weeks. Animals were single-housed following telemetry implantation surgery, with food and water available ad libitum, on a 12 h:12 h (0600–1800) light–dark cycle. The experimental protocol was approved by the Institutional Animal Care and Use Committee at USAMRICD, and all procedures were conducted in accordance with the principles stated in the Guide for the Care and Use of Laboratory Animals [49], the Public Health Service Policy on Humane Care and Use of Laboratory Animals, and the Animal Welfare Act of 1966 (P.L. 89–544), as amended.

### 4.2. Surgeries

A subset of mice were implanted subcutaneously (SC), under 2%–5% isoflurane, at a surgical plane of anesthesia as determined by a lack of response from a strong toe pinch, with ETA-F10, F20-EET, or HDX-02 telemetry transmitters (Data Sciences International; DSI^TM^; St. Paul, MN, USA). Wires were wrapped around cortical stainless steel screws that were placed at 1.5 mm right and/or left of the midline, and 1.5 mm anterior, and 3.0 mm posterior to bregma, as previously described [13]. Meloxicam (Patterson Veterinary, St Paul, MN, USA) was administered at least 30 min prior to surgery (5 mg/kg; SC) and sustained release (SR) buprenorphine (ZooPharm, Windsor, CO, USA) was administered (0.6 mg/kg, SC) immediately after surgery to minimize pain. All mice were given 1–2 weeks of recovery from surgery before exposure.

### 4.3. GD Exposures and Treatments

Mice were exposed SC to either saline (No GD group) or 80 μg/kg GD (pinacolyl methylphosphonofluoridate; United States Army Combat Capabilities Development Command Chemical Biological Center, Aberdeen Proving Ground, Gunpowder, MD, USA), as previously described [13]. Food was removed and cage bedding was replaced with an isopad immediately before exposure. An admix of atropine sulfate (4 mg/kg; Sigma-Aldrich, St Louis, MO, USA) and HI-6 dimethanesulphonate (50 mg/kg; Starkes Associates, Buffalo, NY, USA) was administered intraperitoneally (IP) at 1 min after GD exposure. The therapeutic dose of intramuscular midazolam used in a clinical trial is approximately 0.25 mg/kg [50] and based on body surface area normalization the mouse equivalent dose is estimated to be approximately 3.075 mg/kg, which is close to the dose of midazolam (3 mg/kg; Hospira, Lake Forest, IL, USA) administered SC in Experiment 1 and Experiment 2. In Experimentm 2 ketamine (30 mg/kg; Mylan, Canonsburg, PA, USA) was administered IP.

#### 4.3.1. Experiment 1

For Experiment 1, an initial proof-of-concept study to determine that GD-induced seizure results in neuropathology were performed in female mice since they were more readily available. For experiment 1, female KIKO mice received midazolam (3 mg/kg; SC) mono-therapy at either 15 min after the onset of GD-exposed status epilepticus or at 20 min after saline exposure.

#### 4.3.2. Experiment 2

For Experiment 2, GD-exposed male KIKO and Es1-/- mice were randomly divided into one of two treatment groups consisting of midazolam (3 mg/kg; GD + MDZ; SC) or midazolam combined with ketamine (30 mg/kg; GD + MDZ/KET; IP) at 15 min after seizure onset. Control (No GD) animals received midazolam (3 mg/kg; SC) at 20 min after saline administration.

### 4.4. Behavioral and Electroencephalographic (EEG) Seizure Activity

Following exposure, behavioral signs of toxicity were monitored by an observer blinded to treatment using the Noldus Pocket Observer program (Noldus Information Technology, Wageningen, The Netherlands). Behavioral seizure severity was scored using a modified Racine scale [51] of 6 stages: 0, no abnormality; 1, mastication, tongue fasciculations, oral tonus; 2, head nodding and/or tremors; 3, forelimb clonus or tonus, body tremors; 4, rearing with convulsions; and 5, rearing and falling with convulsions. In instrumented mice, electroencephalographic (EEG) signals were monitored in real-time to determine the onset of seizure activity, defined as the appearance of rhythmic high-amplitude spikes (>2 × baseline) that lasted at least 10 s (based on Nissinen et al. [52]). Electroencephalographic activity was continuously recorded using Dataquest Art Acquisition software (DSI) from three days before exposure up until euthanasia. In non-instrumented mice, the onset of seizure was marked by the appearance of whole-body tremors while walking rapidly (a.k.a. running seizure), which typically occurs just prior to EEG seizure onset. A subset of animals was euthanized at shorter or longer time points than 14 days following GD-induced seizure onset and they were not included in the analysis of SRS onset and the total number of SRS events.

### 4.5. Brain Tissue Collection and Immunohistochemistry

A subset of mice that survived to study endpoint (24 h or 14 days after exposure) were injected with sodium pentobarbital (75 mg/kg, IP, Fatal Plus; Patterson Veterinary), anesthesia was confirmed by lack of a response from a strong toe pinch, and the animals were perfused with heparinized 0.9% saline in 0.1 M phosphate buffer (FD Neurotechnologies, Columbia, MD, USA) followed by a 4% paraformaldehyde solution as previously described [13]. Brains were removed, kept in 4% paraformaldehyde for 6 h, and cryoprotected in 20% sucrose. Sectioning and staining of tissue were performed by FD Neurotechnologies using previously described methods [53]. Frozen brains were coronally cut at a thickness of 30 µm, and stained with Fluoro-Jade B, to visualize dying neurons, and the ionized calcium-binding adaptor molecule 1 (Iba1; rabbit anti-Iba1 IgG 1:6000; Wako Chemicals, Richmond, VA, USA) for experiment 1, or for experiment 2, immunohistochemically processed using antibodies against the neuronal nuclear protein (NeuN; mouse anti-NeuN IgG 1:600; Millipore, Billerica, MA, USA) and Iba1. For Iba1-stained tissue, cresyl violet was used as a counterstain for the visualization of anatomic landmarks.

### 4.6. Cell Counts

Coverslip-mounted, immunostained brain slices were scanned with a 0.40 NA 10X (NeuN and Fluoro Jade B) objective or a 0.75 NA 20X (Iba1) objective using an Olympus BX61IVS microscope with a Pike F-505 camera (Allied Vision, Exton, PA, USA). Image-Pro Plus (Media Cybernetics, Inc., Rockville, MD, USA) was used to trace regions of interest in images and obtain counts of Fluoro-Jade B-positive and NeuN-positive cells using the particle analysis function by a scorer that was blind to treatment groups. Brain regions evaluated included the lateral thalamus, medial thalamus, CA1 region of the hippocampus, basolateral amygdala, and layer 3 of the piriform cortex. For each brain tissue slice, entire brain regions were traced using anatomic landmarks in the region between −1.06 mm to −1.94 mm from bregma. Stereology was performed using the Stereo Investigator software (MBF Bioscience, Williston, VT, USA) to quantify highly dense NeuN-positive neurons in the CA1 region of the hippocampus; five sections per mouse were analyzed using the optical fractionator method in the region from −1.22 to −3.88 mm from bregma [27]. Iba1 is expressed by both active and resting microglia. Therefore, analysis of the density and morphology (i.e., cell-body-to-cell-size ratio, an indication of microglial cell activation [54,55]) of Iba1-positive cells was performed in brain regions using the ImageJ software (National Institutes of Health, Bethesda, MD, USA); methods for quantification of cell morphology were modified, as previously described [13], from published analyses [54,55].

### 4.7. Data Analysis

SPSS version 22 (IBM) was used for all statistical analyses. In the model development experiments, a Student’s *t*-test was used to compare the effect of GD (GD versus no GD (saline control)) on Fluoro-Jade B and Iba1 (cell-body-to-cell-size ratio and density). In the experiments that evaluated the efficacy of ketamine as an adjunct to midazolam treatment, a Kaplan-Meier analysis was performed to estimate and compare treatment effects on median survival time, followed by logistic regression analysis and Chi-square, followed by a Fisher’s exact test for group comparisons of endpoint percent of survival. A Student’s *t*-test was used to compare the effect of midazolam mono-therapy treatment versus midazolam/ketamine dual-therapy on seizure duration, while a one-way analysis of variance (ANOVA) was used to compare the effects of treatment on NeuN and Iba1 cell density, and cell body size–cell size ratio of Iba1-immunoreactive cells; Tukey’s test was used for group comparisons. Differences were considered statistically significant when *p* < 0.05.

## 5. Conclusions

In summary, the present study accomplished the development of a GD exposure model that resulted in severe seizures, neuropathology and epileptogenesis in the novel KIKO mouse model, and further supports its use as an innovative tool to screen for medical countermeasures against the effects of acute CWNA exposure. Moreover, our findings of an increase in survival, reduction in seizure severity, and reduction in neuropathology by a delayed treatment of midazolam/ketamine dual-therapy provide solid evidence that supports the benefits of combining ketamine with a benzodiazepine for the treatment of CWNA-induced status epilepticus.

## Data Availability

The data presented in this study may be available on request from the corresponding author. The data are not publicly available in a repository at this time but may be requested.

## Supplementary Materials

The following are available online at https://www.mdpi.com/1422-0067/22/4/1893/s1, Figure S1: Effect of delayed midazolam treatment combined with ketamine on survival following GD-induced status epilepticus in Es1-/- mice. Figure S2: Effect of ketamine as an adjunct to delayed midazolam treatment on seizure activity and severity in soman-exposed Es1-/- mice.
