# Supplementary material for: Novel Genetically Modified Mouse Model to Assess Soman-Induced Toxicity and Medical Countermeasure Efficacy: Human Acetylcholinesterase Knock-in Serum Carboxylesterase Knockout Mice
Source: Int J Mol Sci. 2021 Feb 14;22(4):1893. doi: 10.3390/ijms22041893 (PMC7918218; doi:10.3390/ijms22041893)
Supplement: Supplementary file 1 [file ijms-22-01893-s001.pdf]

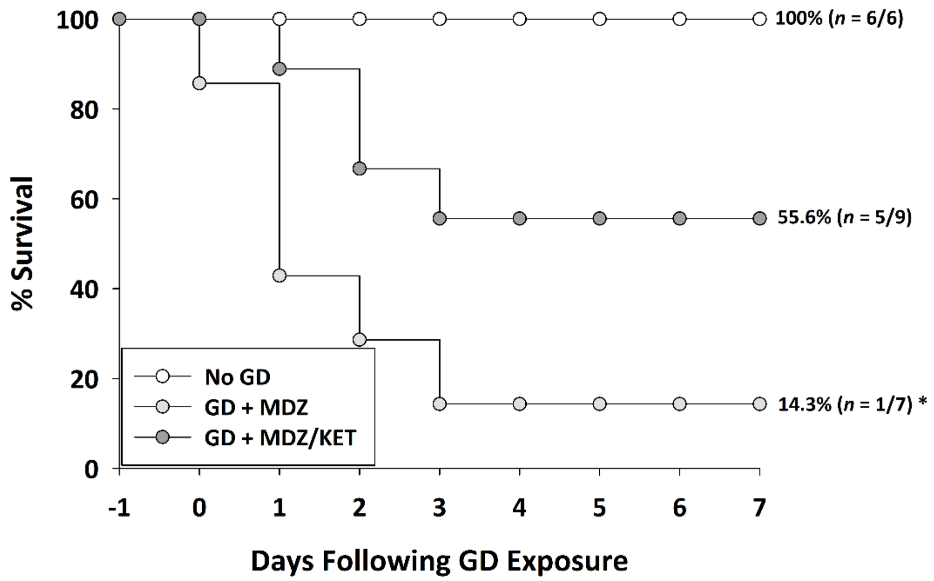

Figure S1. Effect of delayed midazolam treatment combined with ketamine on survival following GD-induced status epilepticus in Es1-/- mice. Mice exposed to GD and treated with midazolam monotherapy (GD + MDZ) at 15 min after seizure onset had a significantly lower percentage of survival compared to that of the saline control (No GD) group. In contrast, soman-exposed mice that were treated with a combination of midazolam and ketamine (GD + MDZ/KET) had a higher percentage of survival that was not significantly different from the percentage of survival of the No GD group. * $p < 0.05$, compared to No GD group.

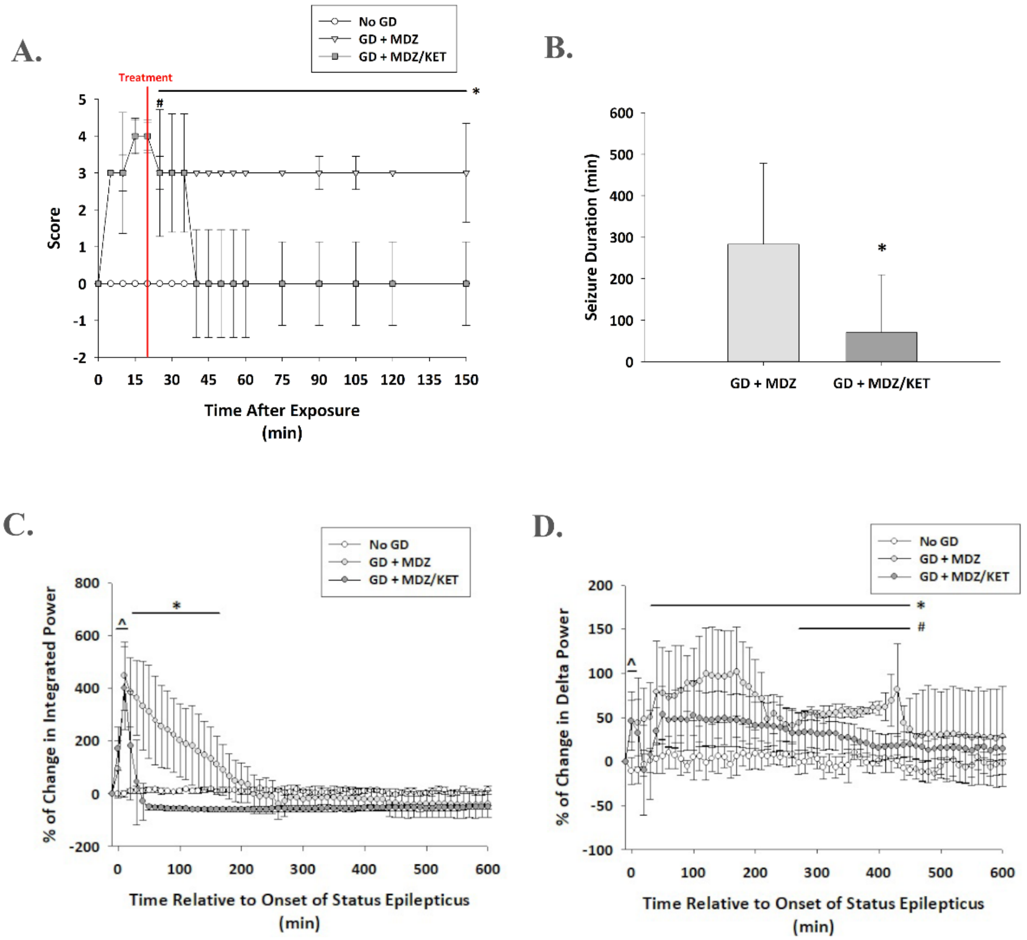

Figure S2. Effect of ketamine as an adjunct to delayed midazolam treatment on seizure activity and severity in soman-exposed Es1-/- mice. (**A**) SC exposure to 80 µg/kg of GD resulted in the appearance of behavioral seizure signs within 5 min of exposure. The severity of toxic signs was scored following a modified Racine scale: 0, no abnormality; 1, mastication, tongue fasciculations, oral tonus; 2, head nodding and/or tremors; 3, forelimb clonus or tonus, body tremors; 4, rearing with convulsions; and 5, rearing and falling with convulsions. Toxic signs for the midazolam/ketamine group (GD + MDZ/KET; $n = 8$) were transiently reduced in severity within 10 min of treatment (indicated by a red line; 15 min after EEG seizure onset). In contrast, toxic signs in the midazolam monotherapy group (GD + MDZ; $n = 6$) did not subside. * $p < 0.05$, GD + MDZ compared to no agent control (No GD; $n = 6$); # $p < 0.05$, GD + MDZ/KET compared to No GD. (**B**) Soman exposure elicited seizure that had an average duration of (± SD) 283.2 ± 195.8 min in the GD + MDZ group, while an average of 70.5 ± 139.2 min was observed in the GD + MDZ/KET group. Tracings of average percentages of relative change in (**C**) integrated power (freq. range) and (**D**) delta (0.1–4 Hz) EEG frequency are shown over a period of 600 min (10 h). In Es1-/- mice, the midazolam/ketamine combination therapy was able to reduce over time the increase in integral power and delta power immediately following administration of treatment at 15 min following onset of status epilepticus. * $p < 0.05$, GD + MDZ ($n = 6$) compared to No GD ($n = 5$) group; ^ $p < 0.05$, GD + MDZ/KET ($n = 8$) compared to No GD group.
